# Lysophosphatidic Acid Induces Apoptosis of PC12 Cells Through LPA1 Receptor/LPA2 Receptor/MAPK Signaling Pathway

**DOI:** 10.3389/fnmol.2020.00016

**Published:** 2020-02-06

**Authors:** Jie Zhang, Yiyi Li, Chao Wang, Yaya Wang, Yangyang Zhang, Liqin Huang, Zhaohui Zhang

**Affiliations:** Department of Neurology, Renmin Hospital of Wuhan University, Wuhan, China

**Keywords:** LPA, LPA1, LPA2, MAPK, apoptosis, mitochondrial dysfunction

## Abstract

Lysophosphatidic acid is a small extracellular signaling molecule, which is elevated in pathological conditions such as ischemic stroke and traumatic brain injury (TBI). LPA regulates the survival of neurons in various diseases. However, the molecular mechanisms underlying LPA-induced neuronal death remain unclear. Here we report that LPA activates LPA1 and LPA2 receptors, and the downstream MAPK pathway to induce the apoptosis of PC12 cells through mitochondrial dysfunction. LPA elicits the activation of ERK1/2, p38, and JNK pathways, decreases the expression of Bcl2, promotes the translocation of Bax, and enhances the activation of caspase-3, resulting in mitochondrial dysfunction and cell apoptosis. This process can be blocked by LPA1 receptor antagonist and LPA2 receptor antagonist and MAPK pathway inhibitors. Our results indicate that LPA1 receptor, LPA2 receptor and MAPK pathway play a critical role in LPA-induced neuronal injury. LPA receptors and MAPK pathways may be novel therapeutic targets for ischemic stroke and TBI, where excessive LPA signaling exist.

## Introduction

Lysophosphatidic acid (LPA) is the structurally simplest phospholipid, which functions as an extracellular signaling molecule via binding to its receptors. Six G protein-coupled LPA receptors have been reported (Hecht et al., 1996; Noguchi et al., 2003; Kotarsky et al., 2006; Lee et al., 2006; Pasternack et al., 2008; Wu et al., 2018). Activation of LPA receptors results in the activation of various downstream pathways including MAPK, Rock, and PI3K (Wu et al., 2018). Through activating these pathways, LPA mediates a series of cellular functions including cell proliferation, cell migration, and cell survival (Wu et al., 2018).

In recent years, LPA has been found to induce neuronal death both *in vitro* and *in vivo* (Ramesh et al., 2018). Steiner et al. (2000) found that micromole of LPA induced cell death of cultured hippocampal neurons and neuronal PC12 cells. The concentration of LPA in the cerebrospinal fluid of patients with traumatic brain injury (TBI) was elevated compared to controls. The administration of an LPA monoclonal antibody blocked LPA signaling and exerted a protective effect against TBI-induced brain injury (Crack et al., 2014). Wang et al. (2018), we reported that after ischemic brain injury, the concentration of LPA was increased in the rat brain, while an inhibitor of autotaxin, which is the enzyme that catalyzes the production of LPA, reduced the apoptotic rate of neurons in a rat model of ischemia-induced brain injury. These evidence suggest that LPA may regulate neuronal damage under pathological conditions. However, the specific molecular mechanisms underlying LPA-induced neuronal death remains unclear.

Our current research focuses on how LPA induces neuronal apoptosis as it is one of the major pathways by which neuronal death occurs (Hollville et al., 2019). Neuronal apoptosis induced by LPA was accompanied by a decrease in mitochondrial membrane potential (MMP) (Steiner et al., 2000), hence we tested whether mitochondrial dysfunction contributes to LPA-induced cell death. Among the six LPA receptors, LPA1 receptor and LPA2 receptor are most extensively studied and their specific antagonists are widely used in research (Lee et al., 2019; Lopez-Serrano et al., 2019). It has been reported that LPA induces activation of the MAPK pathway, which consists of ERK1/2, p38, and JNK (Park et al., 2018). Here we investigated the molecular mechanisms underlying LPA-induced cell death. We found that the activation of LPA1 receptor/LPA2 receptor/MAPK pathway and mitochondrial dysfunction contribute to LPA-induced neuronal injury.

## Materials and Methods

### Materials

RevertAid First Strand cDNA Synthesis Kit was purchased from Thermo Fisher, Inc. qPCR SYBR Green Master Mix (Q141) was purchased from Vazyme. 18:1 LPA (L7260) was purchased from Sigma-Aldrich. One step TUNEL apoptosis assay kit (C1080), cell mitochondria isolation kit (C3601), and Rhodamine123 (C2007) were purchased from Beyotime. Anti-Bcl2 antibody (ab182858), anti-Bax antibody (ab32503), anti-p-JNK antibody (ab124956), and anti-JNK antibody (ab179461) were purchased from Abcam. Anti-p-ERK1/2 antibody (AP0472), anti-ERK1/2 antibody (A10613), anti-p-p38 antibody (AP0056), and anti-p38 antibody (A10832) were purchased from ABclonal. Anti-GAPDH antibody (10494-1-AP), anti-cleaved caspase-3 antibody (66470-2-Ig), and anti-COXIV antibody (11242-1-AP) were purchased from Proteintech. Cell Counting Kit-8 (CCK-8, HY-K0301), LPA1 receptor antagonist (AM095, HY-16039; BMS986020, HY-100619), LPA2 receptor antagonist (HY-18075), ERK1/2 inhibitor (U0126, HY-12031), p38 inhibitor (SB203580, HY-10256), and JNK inhibitor (SP600125, HY-12041) were purchased from MedChemExpress.

### Cell Culture

PC12 cells were obtained from the China Center for Type Culture Collection, and cultured in DMEM medium supplemented with 10% horse serum (v/v), 5% fetal bovine serum (v/v), 50 U/ml penicillin, and 50 μg/mL streptomycin at 37°C in a 5% CO_2_ humidified incubator. The cells were differentiated by incubation in DMEM medium supplemented with 50 ng/ml nerve growth factor for 2 days before experiments. Primary rat cortical neurons were prepared from E16 embryos, and cultured as previously described (Liu et al., 2008; Meng et al., 2019) at 37°C at 37°C in a 5% CO_2_ humidified incubator. Cortical neurons were cultured for 8 days *in vitro* before experiments.

### Preparation of LPA Stock Solutions and Treatment of Cells

Lysophosphatidic acid stock solution was prepared by dissolving LPA in calcium- and magnesium-free phosphate buffered saline (PBS), pH 7.2 in the presence of 1% (w/v) bovine serum albumin (essentially fatty acid-free). 5 mg LPA is dissolved in about 11.45 ml PBS, and 1 mM LPA stock solutions is achieved.

Neuronal PC12 cells were incubated in Locke’s solution before LPA treatment and were treated in three ways. The first way of treatment: PC12 cells were treated with different concentrations of LPA (20, 40, 60 μM, respectively)/BSA or BSA alone for 24 h and then were used for further experiments. The second way of treatment: PC12 cells were treated with LPA/BSA for various time (0, 6, 12, and 24 h, respectively), and were used for further experiments. The third way of treatment: PC12 cells were pretreated with DMSO (vehicle), LPA1 receptor antagonist (AM095, 5 μM), LPA2 receptor antagonist (5 μM), ERK1/2 inhibitor (U0126, 5 μM), p38 inhibitor (SB203580, 10 μM), or JNK inhibitor (SP600125, 10 μM) for 2 h, then all groups were subjected to LPA for 24 h. The cells were used for further experiments.

Primary neurons were cultured as previously described (Wang et al., 2018). The neurons were treated with LPA in the presence of LPA1 receptor antagonist (AM095, 5 μM), LPA2 receptor antagonist (5 μM), ERK1/2 inhibitor (U0126, 5 μM), p38 inhibitor (SB203580, 10 μM), or JNK inhibitor (SP600125, 10 μM), and then were used for further experiments.

### CCK-8 Assay

The viability of neuronal PC12 cells was measured using the CCK-8 assay kit according to the manufacture’s manual. Briefly, PC12 cells were plated into a 96-well plate (10000 cells/well) and cultured in the presence of LPA for the desired time. 10 μl CCK-8 solution was mixed with 100 μl medium and added to each well. The absorbance at 450 nm was measured after 2 h of treatment.

### TUNEL Staining

Apoptotic DNA fragmentation was examined using the One-step TUNEL apoptosis assay kit according to the manufacture’s protocol. Briefly, PC12 cells were plated into a 24-well plate and cultured in the differentiation medium for 48 h, and then were treated as described (see section “CCK-8 Assay”). The cells were fixed in 4% paraformaldehyde for 30 min, permeabilized in 0.3% Triton X-100 for 5 min, and then incubated with TUNEL kits for 1 h at 37°C. The slides were washed with PBS, and stained with DAPI solution for 5 min. Four independent fields were selected for examination. The percentage of TUNEL-positive nuclei in the region was calculated to evaluate apoptosis. The marker index is measured by the number of dead cells per visual field/all the cells in the visual field, and the apoptotic index (AI) of each sample was equal to the mean value of marker index in different visual fields.

### Measurement of Mitochondrial Membrane Potential (ΔΨm)

Mitochondrial membrane potential was assessed by Rhodamine 123 (Rh123) probe. PC12 cells were plated into 6-well plates. After treatment, the cells were incubated with 5 μM Rh123 at 37°C for 30 min. Then the fluorescence intensity of Rh123 was measured by a fluorescence microscope or flow cytometry. The depolarization of MMP causes a rise in fluorescence intensity of Rh123 (Huang et al., 2007).

### Quantitative PCR

PC12 cells were plated into culture dishes (6 cm in diameter) and cultured in the differentiation medium for 48 h, and then were treated as described. Total RNA was extracted using Trizol reagent (Thermo Fisher). cDNA was synthesized using the RevertAid First Strand cDNA Synthesis Kit (Thermo Fisher). SYBR Green Master Mix was used to perform real-time PCR. The primers used were as follows: Bcl2 forward primer AGCATGCGACCTCTGTTTGA, Bcl2 reverse primer TCACTTGTGGCCCAGGTATG; GAPDH forward primer AC GGGAAGCTCACTGGCATGG, GAPDH reverse primer CGCCTGCTTCACCACCTTCTT. The PCR procedure was as follows: Pre-incubation: 95°C 30 s for 1 cycle; PCR: 95°C 5 s, 56°C 30 s, 72°C 30 s, for 40 cycles; Melting: 95°C 1 s, 65°C 15 s, for 1 Cycle.

### Western Blot

The cells were harvested and lysed in lysis buffer (50 mM Tris, pH 7.4, 40 mM NaCl, 1 mM EDTA, 0.5% Triton X-100, 1.5 mM Na_3_VO_4_, 50 mM NaF, 10 mM sodium pyrophosphate, 10 mM sodium β-glycerophosphate, supplemented with protease inhibitors cocktail). To extract the mitochondrial proteins, the cells washed with PBS and centrifuged at 600 *g* for 5 min. The pallet was resuspended in 500 μl mitochondrial extraction buffer, and incubated on ice for 15 min. Then the cells were lyzed in a glass homogenizer. The solutions were then centrifuged at 600 *g* for 10 min. 60 μl mitochondrial lysis buffer was added to the pallet and incubated for 15 min on ice. The protein concentrations were determined using BCA kit. An equal amount of 15 μg protein was loaded among samples.

Western blot was implemented as described previously (Ning et al., 2004). Briefly, the proteins were transferred to PVDF membranes (Millipore). 5% non-fat milk or BSA in PBS was applied to block the membranes at room temperature for 1.5 h. The membranes were then incubated with the following antibodies: Anti-Bcl2 antibody (1:1000), anti-Bax antibody (1:2000), anti-p-ERK1/2 antibody (1:1000), anti-ERK1/2 antibody (1:1000), anti-p-p38 antibody (1:1000), anti-p38 antibody (1:1000), anti-p-JNK antibody (1:500), anti-JNK antibody (1:1000), anti-cleaved caspase-3 antibody (1:500), anti-GAPDH antibody (1:20000), or anti-COXIV antibody (1:5000) overnight at 4°C. The membranes were then incubated with the appropriate horseradish peroxidase-conjugated secondary antibody (1:5000) at room temperature for 1.5 h. The protein bands were detected with ECL Western Blotting Substrate Kit.

### Statistical Analysis

All the quantitative data were presented as mean ± SEM. Statistical analysis was performed using Mann–Whitney *U*-test or Kruskal–Wallis test followed by Bonferroni *post hoc* test. Differences with *P*-values < 0.05 were considered significant.

## Results

### LPA Induces Apoptosis and Mitochondrial Dysfunction in Neuronal PC12 Cells and Primary Neurons

**FIGURE 1:**
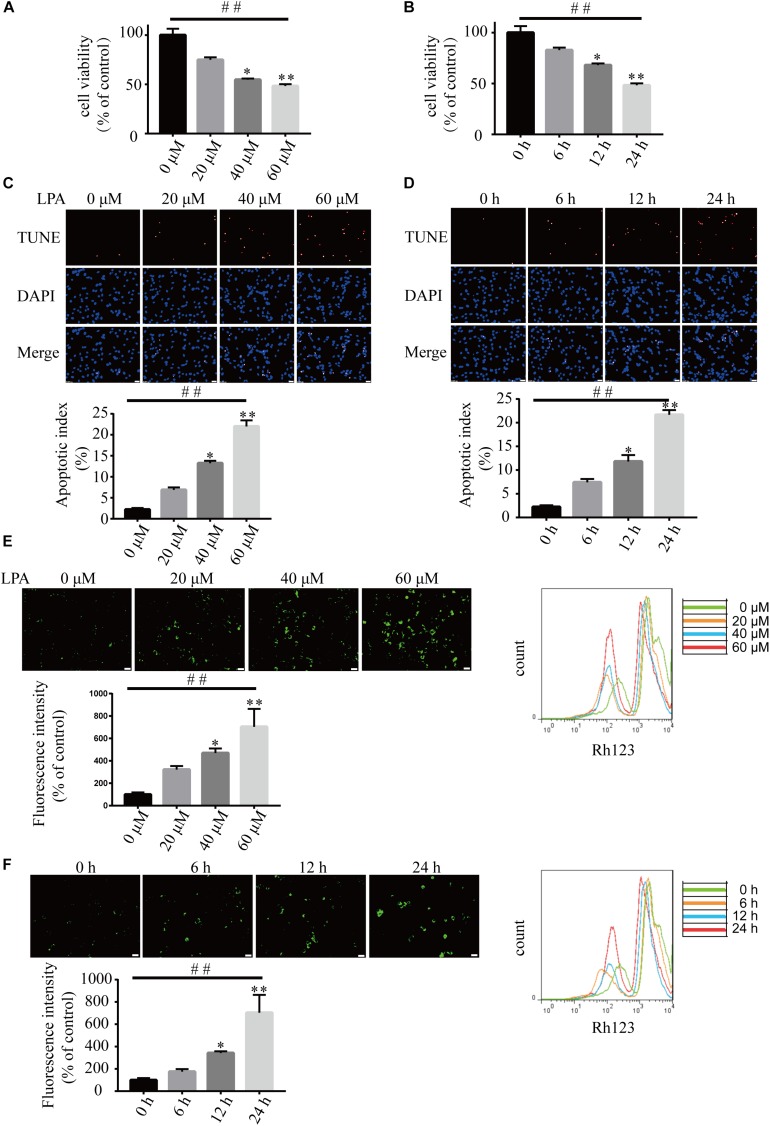
Lysophosphatidic acid (LPA) induces cell injury and mitochondrial dysfunction in a time- and dose-dependent manner. CCK-8 measures the viability of neuronal PC12 cells treated with LPA **(A,B)**. TUNEL staining detects the apoptosis of neuronal PC12 cells following treatment **(C,D)**. Rh123 staining estimates the MMP of neuronal PC12 cells after treatment **(E,F)**. Scale bar: 20 μm. Data are mean ± SEM of five independent experiments for Kruskal–Wallis test, ##*P* < 0.01; for Bonferroni *post hoc* test (compare each group with control), ^∗^*P* < 0.05, ^∗∗^*P* < 0.01.

We first examined the effect of LPA on cell viability using CCK-8 kit. LPA decreased the cell viability of neuronal PC12 cells in a concentration- and time-dependent manner (Figures 1A,B). TUNEL staining found that LPA increased the apoptotic rate of PC12 cells in a concentration- and time-dependent manner (Figures 1C,D). Next, we detected the MMP of neuronal PC12 cells treated with LPA using Rh123 staining. Fluorescence microscopy and flow cytometry experiments found that LPA induced a significant increase in fluorescence intensity of Rh123 in a concentration- and time-dependent manner (Figures 1E,F). To confirm the effect of LPA, we also tested the effect of LPA in primary neurons and got similar results as in neuronal PC12 cells (Supplementary Figures S1a,b). These results indicate that the LPA induces cell injury and loss of MMP impairments both in neuronal PC12 cells and cultured neurons.

**FIGURE 2:**
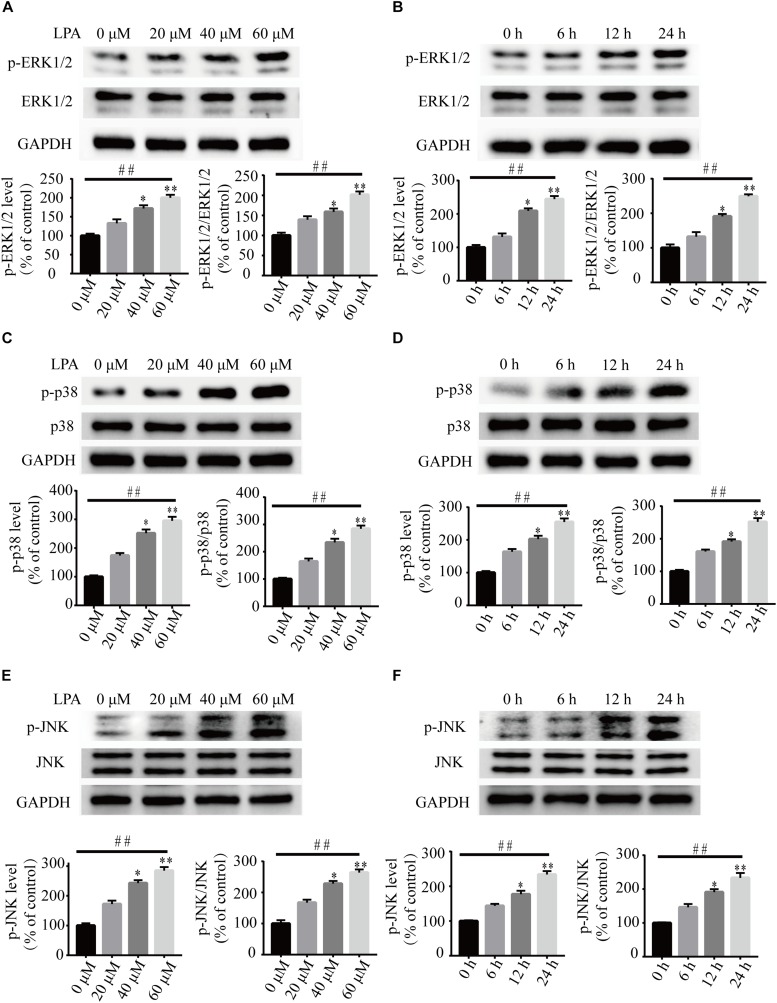
The activation of ERK1/2, p38, and JNK pathways induced by LPA. Western blot showing the activation of ERK1/2 **(A,B)**, p38 **(C,D)**, and JNK pathway **(E,F)** in neuronal PC12 cells treated with LPA. Data are mean ± SEM of five independent experiments for Kruskal–Wallis test, ##*P* < 0.01; for Bonferroni *post hoc* test (compare each group with control), ^∗^*P* < 0.05, ^∗∗^*P* < 0.01.

**FIGURE 3:**
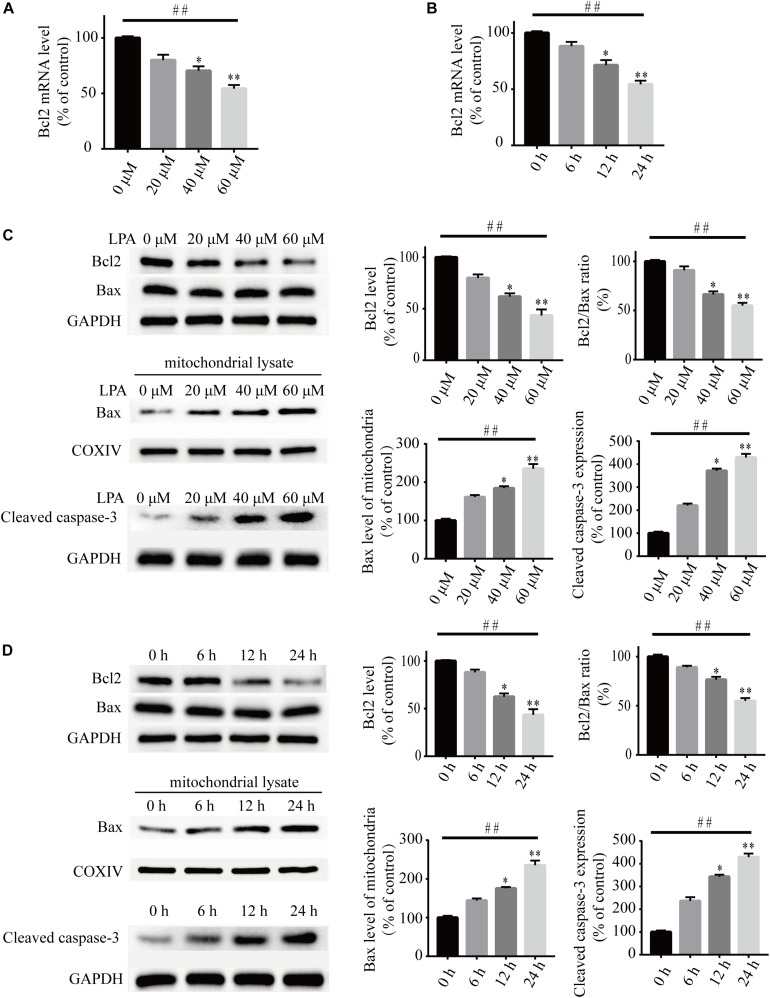
Lysophosphatidic acid decreases the Bcl2 mRNA level, Bcl2 protein level, and Bcl2/Bax ratio while it increases the translocation of Bax and the activation of caspase-3 in neuronal PC12 cells. Q-PCR measures the Bcl2 mRNA level in neuronal PC12 cells treated with LPA **(A,B)**. Western blot detects the protein levels of Bcl2, Bax and cleaved caspase-3 in whole cells as well as the Bax protein level in mitochondria of neuronal PC12 cells **(C,D)**. Data are mean ± SEM of five independent experiments for Kruskal–Wallis test, ##*P* < 0.01; for Bonferroni *post hoc* test (compare each group with control), ^∗^*P* < 0.05, ^∗∗^*P* < 0.01.

To investigate the underlying molecular mechanisms of LPA-induced cell injury, We performed Western blot and quantitative PCR to investigate the expression of MMP-related genes. As shown in Figure 2, LPA challenge elicited ERK1/2, p38 and JNK phosphorylation in a concentration- and time-dependent manner, indicating LPA induces the activation of the MAPK pathways (Figures 2A–F). We further investigated the expression of apoptosis-related genes, and found that Blc2 mRNA level, Bcl2 protein level, and Bcl2/Bax ratio were all significantly decreased in neuronal PC12 cells following LPA treatment (Figures 3A–D). LPA also induced the translocation of Bax from the cytoplasm to mitochondria. The protein level of cleaved caspase-3 was elevated after LPA treatment (Figures 3C,D). These results indicate that LPA activates the MAPK pathways and induces mitochondrial dysfunction.

### Blockade of LPA1 Receptor and LPA2 Receptor Attenuates LPA-Induced Neuronal Damage and Mitochondrial Dysfunction

**FIGURE 4:**
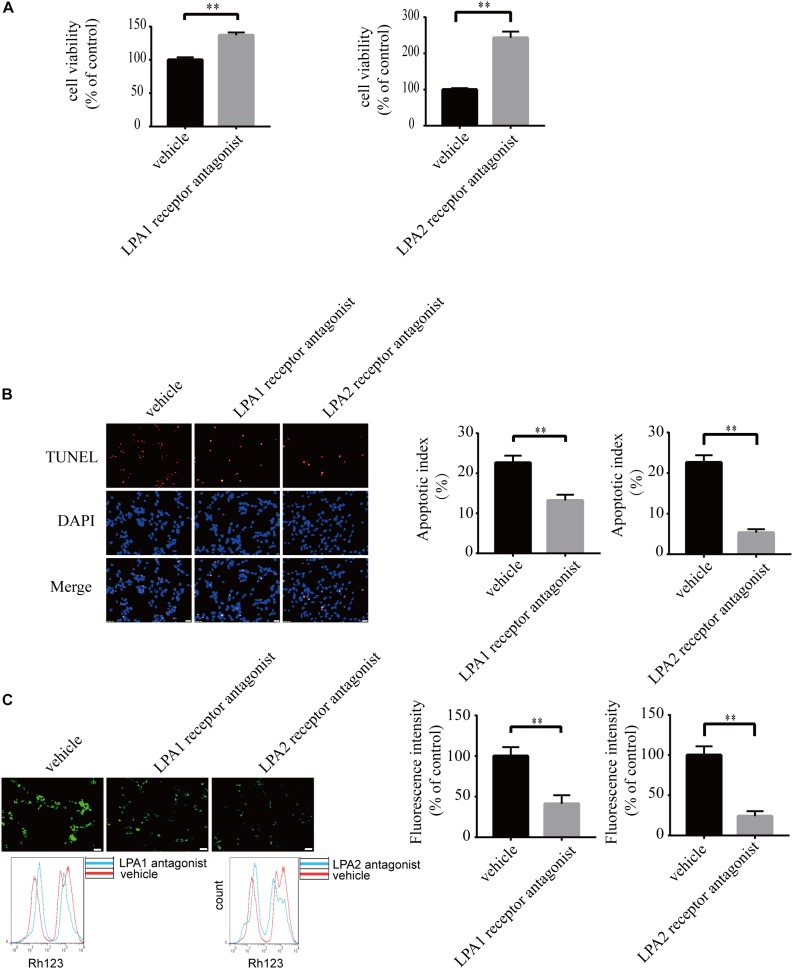
Blockade of LPA1 receptor and LPA2 receptor prevents LPA-induced neuronal damage, and alleviates mitochondrial dysfunction. The viability of neuronal PC12 cells was measured using CCK-8 kit **(A)**. The apoptosis of neuronal PC12 cells was detected by TUNEL staining **(B)**. The MMP of neuronal PC12 cells was estimated by Rh123 staining **(C)**. Scale bar: 20 μm. Data are mean ± SEM of five independent experiments. ^∗∗^*P* < 0.01.

**FIGURE 5:**
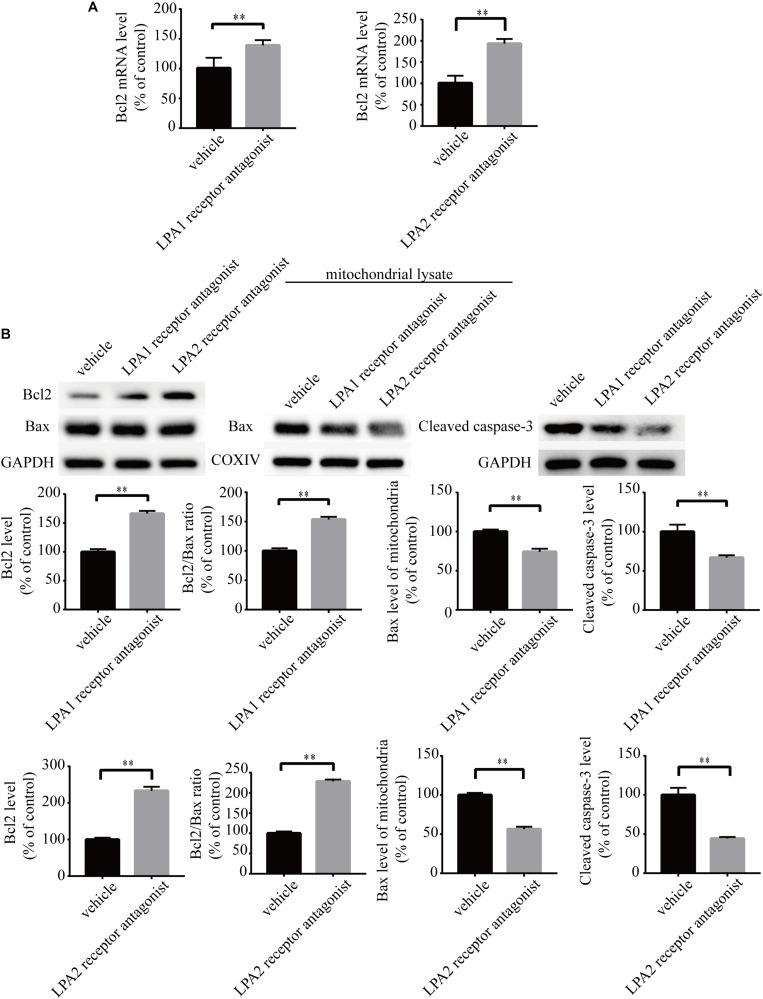
LPA1 receptor antagonist and LPA2 receptor antagonist attenuate the decrease of Bcl2 mRNA level, Bcl2 protein level, and Bcl2/Bax ratio as well as the activation of caspase-3. Q-PCR measures the Bcl2 mRNA level in neuronal PC12 cells treated with LPA **(A)**. Western blot detects the Bcl2, Bax and cleaved caspase-3 protein levels in whole cells as well as the Bax protein level in mitochondria of neuronal PC12 cells **(B)**. Data are mean ± SEM of five independent experiments. ^∗∗^*P* < 0.01.

To explore the roles of LPA1 receptor and LPA2 receptor in LPA-induced neuronal injury, we treated PC12 cells with LPA1 receptor antagonist and LPA2 receptor antagonist, respectively, and then exposed the cells to 60 μM LPA. We found that both LPA1 receptor antagonist and LPA2 antagonist markedly mitigated neuronal injury induced by LPA as shown by CCK-8 and TUNEL staining analysis (Figures 4A,B). MMP analysis demonstrated that both LPA1 receptor antagonist and LPA2 receptor antagonist significantly ameliorated mitochondrial dysfunction induced by LPA (Figure 4C). Moreover, both the LPA1 receptor antagonist and LPA2 receptor antagonist blocked the decrease of Bcl2 mRNA level, Bcl2 protein level, and Bcl2/Bax ratio induced by LPA. LPA1 receptor antagonist and LPA2 receptor antagonist also blocked the translocation of Bax and the activation of caspase-3 (Figures 5A,B). We also assessed the effect of LPA receptor antagonists on cell injury induced by a lower concentration of LPA (20 μM), and got similar results as in cells treated with 60 μM LPA (Supplementary Figures S2a–c). To further confirm the role of LPA receptors in LPA-induced cell injury, we tested the effect of another LPA1 receptor antagonist, BMS986020. BMS986020 exerted similar protective effects as AM095 (Supplementary Figures S3a–c). In primary neurons, we got similar results as in PC12 cells (Supplementary Figures S4a,b). These results indicate that LPA1 receptor and LPA2 receptor mediate LPA-induced neuronal injury and mitochondrial dysfunction.

### Blockade of ERK1/2, p38, and JNK Pathways Ameliorates LPA-Induced Neuronal Injury and Mitochondrial Dysfunction

**FIGURE 6:**
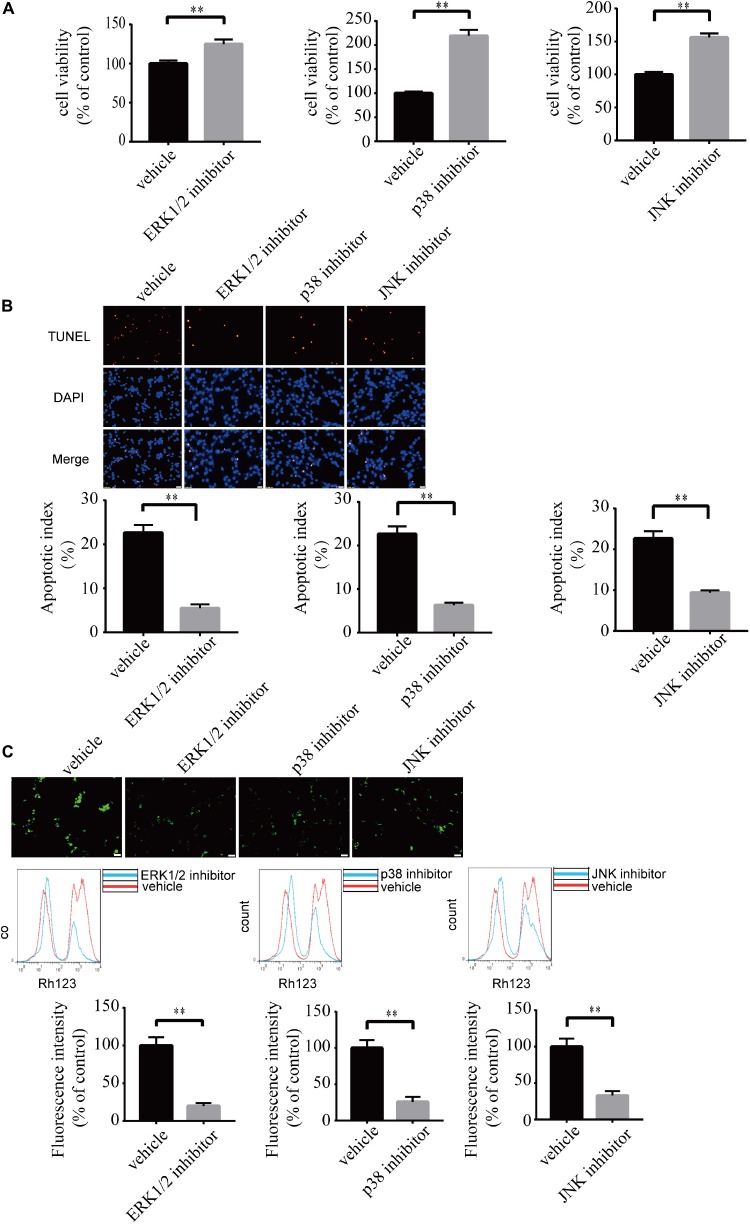
Blockade of MAPK pathway prevents LPA-induced neuronal damage, and alleviates mitochondrial dysfunction. The viability of neuronal PC12 cells was measured using CCK-8 kit **(A)**. The apoptosis of neuronal PC12 cells was detected by TUNEL staining **(B)**. The MMP of neuronal PC12 cells was estimated by Rh123 staining **(C)**. Scale bar: 20 μm. Data are mean ± SEM of five independent experiments. ^∗∗^*P* < 0.01.

**FIGURE 7:**
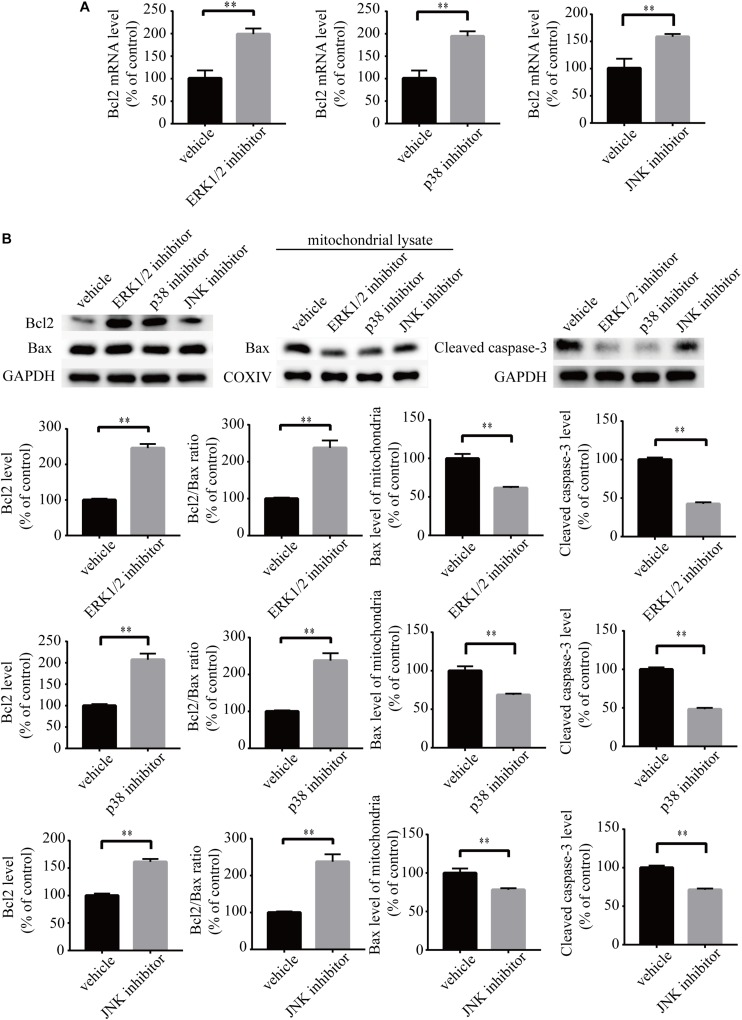
MAPK pathway inhibitors attenuate the decrease of Bcl2 mRNA level, Bcl2 protein level, and Bcl2/Bax ratio as well as the activation of caspase-3. Q-PCR measures the Bcl2 mRNA level in neuronal PC12 cells treated with LPA **(A)**. Western blot detects the Bcl2, Bax, and cleaved caspase-3 protein levels in whole cells as well as the Bax protein level in mitochondria of neuronal PC12 cells **(B)**. Data are mean ± SEM of five independent experiments. ^∗∗^*P* < 0.01.

To investigate the role of MAPK pathways in the toxic effect of LPA, we tested the effect of ERK1/2 inhibitor, p38 inhibitor, and JNK inhibitor on LPA-induced neuronal injury. We pre-incubated the cells with ERK1/2 inhibitor, p38 inhibitor, and JNK inhibitor, respectively, and then added 60 μM LPA to the culture medium. We found that ERK1/2 inhibitor, p38 inhibitor, and JNK inhibitor markedly ameliorated LPA-induced neuronal injury, as shown by CCK-8 and TUNEL staining analysis (Figures 6A,B). Furthermore, the MAPK inhibitors also alleviated the mitochondrial dysfunction induced by LPA as demonstrated by the detection of MMP (Figure 6C). Besides, ERK1/2 inhibitor, p38 inhibitor, and JNK inhibitor blocked the decrease of Bcl2 mRNA level, Bcl2 protein level, and Bcl2/Bax ratio as well as the translocation of Bax and the activation of caspase-3 (Figures 7A,B). We also assessed the effect of the MAPK inhibitors on lower concentration of LPA (20 μM). These inhibitors also attenuated cell injury and mitochondrial dysfunction induced by 20 μM of LPA (Supplementary Figures S5a–c). In primary neurons, we also got similar results as in PC12 cells (Supplementary Figures S6a,b). These results indicate that ERK1/2, p38 and JNK pathways participate in LPA-induced neuronal damage and mitochondrial dysfunction.

## Discussion

Apoptosis is a form of programed cell death. It is mediated through endogenous and exogenous pathways (Zaman et al., 2014; Green and Llambi, 2015). Mitochondrial dysfunction plays a pivotal role in the endogenous apoptotic pathway (Lopez and Tait, 2015). It has been reported that the relative ratio of anti-apoptotic protein Bcl2 and pro-apoptotic protein Bax decides the fate of cells. When the ratio of Bcl2/Bax decreases, Bax translocates from the cytoplasm to mitochondria, which contributes to mitochondrial dysfunction (Huang et al., 2019). Following mitochondrial dysfunction, downstream apoptosis-related protein caspase-3 IS activated, which led to cell apoptosis (Martinou and Youle, 2011; Hassan et al., 2014; Green and Llambi, 2015).

In the present study, LPA-induced cell apoptosis was accompanied by decreased MMP, an observation that is consistent with the findings by Steiner et al. (2000). More importantly, we found that LPA induced a reduction in Bcl2 mRNA levels, Bcl2 protein levels as well as the ratio of Bcl2/Bax. Consequently, the translocation of Bax from the cytoplasm to mitochondria was increased, and apoptosis-related protein caspase-3 was activated. Furthermore, we demonstrated that this process was mediated by LPA1 receptor, LPA2 receptor, and MAPK pathways, as the pathological process of LPA was blocked by LPA1 receptor antagonist, LPA2 receptor antagonist, and MAPK pathway inhibitors.

Previous studies found that LPA and MAPK pathways mediate distinct changes in different cells. For example, LPA induces the proliferation of ovarian carcinoma cells (Rogers et al., 2018). However, LPA induces cell apoptosis in neurons (Steiner et al., 2000). Lower concentrations of LPA (0.1–1 μM) has been reported to attenuate apoptosis induced by Lipopolysaccharide (LPS) in human umbilical cord mesenchymal stem cells. However, LPA at high concentrations (>1 μM) induces cell injury (Li et al., 2017). These results indicate that the effect of LPA is concentration-dependent. Here we show that LPA induced cell injury in PC12 cells and primary neurons. These results suggest that the effect of LPA is dependent on its concentration and the cell type.

Ischemic stroke and TBI induce cell apoptosis and mitochondrial dysfunction (Cheng et al., 2012; Yang et al., 2018; Han et al., 2019). Here we show that LPA induced cell apoptosis and mitochondrial dysfunction, which was blocked by LPA1 receptor antagonist, LPA2 receptor antagonist, and MAPK pathway inhibitors. Since the LPA level in the brain increased after ischemic stroke and TBI, and the administration of LPA-directed monoclonal antibody and autotaxin inhibitor reversed the neuronal damage, we believe that LPA/LPA receptors/MAPK axis plays an important role in ischemic stroke and TBI (Li et al., 2008; Crack et al., 2014; Wang et al., 2018).

In summary, our results indicate that LPA1 receptor/LPA2 receptor/MAPK pathway and mitochondrial dysfunction mediate the neuronal apoptosis induced by LPA. The LPA1 receptor antagonist, LPA2 receptor antagonist, and inhibitors against MAPK pathways may be novel therapeutic strategies for patients with diseases like ischemic stroke and TBI, where excessive LPA signaling exist.

## Data Availability Statement

All datasets generated for this study are included in the article/Supplementary Material.

## Author Contributions

JZ and YL performed most of the experiments. ZZ conceived the project and designed the experiments. CW, YW, YZ, and LH participated in data analysis. All authors have contributed to this last version of the manuscript.

## Conflict of Interest

The authors declare that the research was conducted in the absence of any commercial or financial relationships that could be construed as a potential conflict of interest.

## Supplementary Material

FIGURE S1 Lysophosphatidic acid induces cell injury and mitochondrial dysfunction in a dose-dependent manner. TUNEL staining detects the apoptosis of primary neurons following treatment **(a)**. Rh123 staining estimates the MMP of primary neurons after treatment **(b)**. Scale bar: 20 μm. Data are mean ± SEM of four independent experiments for Kruskal–Wallis test, ##*P* < 0.01; for Bonferroni *post hoc* test (compare each group with control), ^∗^*P* < 0.05, ^∗∗^*P* < 0.01.

FIGURE S2 Blockade of LPA1 receptor and LPA2 receptor prevent LPA-induced neuronal damage as well as alleviating mitochondrial dysfunction. The viability of neuronal PC12 cells was measured with CCK-8 following treatment **(a)**. The apoptosis of neuronal PC12 cells was detected by TUNEL staining after treatment. Scale bar: 50 μm. **(b)** The MMP of neuronal PC12 cells was estimated by Rh123 staining following treatment. Scale bar: 20 μm. **(c)** Data are mean ± SEM of four independent experiments. ^∗^*P* < 0.05, ^∗∗^*P* < 0.01.

FIGURE S3 Blockade of LPA1 receptor using BMS986020 prevents LPA-induced neuronal damage and alleviates mitochondrial dysfunction. The viability of neuronal PC12 cells was measured using CCK-8 kit **(a)**. The apoptosis of neuronal PC12 cells was detected by TUNEL staining. Scale bar: 20 μm. **(b)** The MMP of neuronal PC12 cells was estimated by Rh123 staining. Scale bar: 50 μm. **(c)** Data are mean ± SEM of four independent experiments. ^∗^*P* < 0.05, ^∗∗^*P* < 0.01.

FIGURE S4 Blockade of LPA1 receptor and LPA2 receptor protects against LPA-induced neuronal damage, and alleviates mitochondrial dysfunction. The apoptosis of primary neurons was detected by TUNEL staining **(a)**. The MMP of neuronal primary neurons was estimated by Rh123 staining **(b)**. Scale bar: 20 μm. Data are mean ± SEM of four independent experiments. ^∗^*P* < 0.05, ^∗∗^*P* < 0.01.

FIGURE S5 Blockade of MAPK pathway prevent LPA-induced neuronal damage as well as alleviating mitochondrial dysfunction. The viability of neuronal PC12 cells was measured with CCK-8 following treatment **(a)**. The apoptosis of neuronal PC12 cells was detected by TUNEL staining after treatment. Scale bar: 50 μm **(b)**. The MMP of neuronal PC12 cells was estimated by Rh123 staining following treatment. Scale bar: 20 μm. **(c)** Data are mean ± SEM of four independent experiments. ^∗^*P* < 0.05, ^∗∗^*P* < 0.01.

FIGURE S6 Blockade of MAPK pathway protects against LPA-induced neuronal damage, and alleviates mitochondrial dysfunction. The apoptosis of primary neurons was detected by TUNEL staining **(A)**. The MMP of neuronal primary neurons was estimated by Rh123 staining **(B)**. Scale bar: 20 μm. Data are mean ± SEM of four independent experiments. ^∗^*P* < 0.05, ^∗∗^*P* < 0.01.
