# Supplementary figures and images for: Lysophosphatidic Acid Induces Apoptosis of PC12 Cells Through LPA1 Receptor/LPA2 Receptor/MAPK Signaling Pathway
Source: Front Mol Neurosci. 2020 Feb 6;13:16. doi: 10.3389/fnmol.2020.00016 (PMC7016214; doi:10.3389/fnmol.2020.00016)

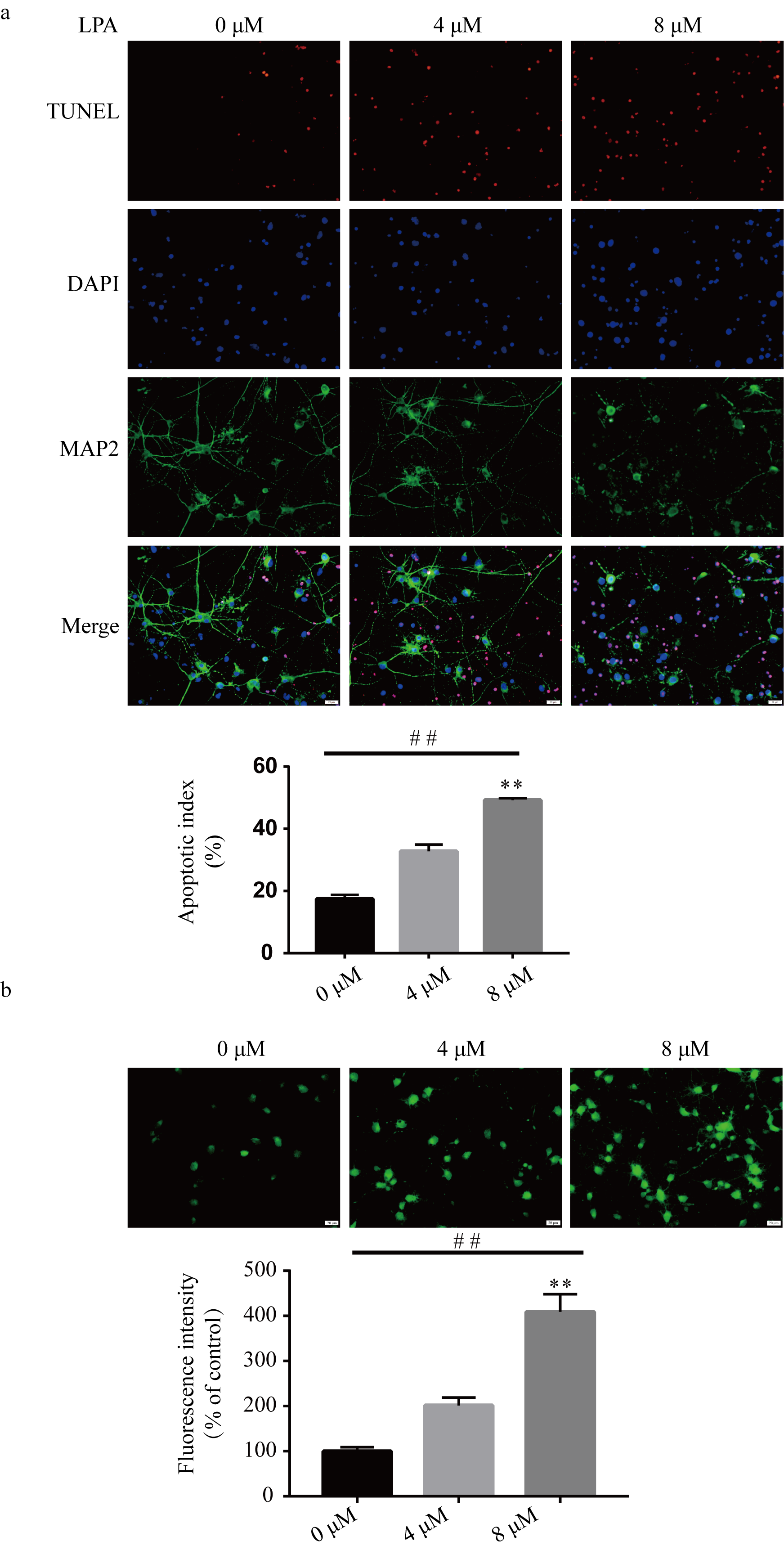

Supplement: FIGURE S1 — Lysophosphatidic acid induces cell injury and mitochondrial dysfunction in a dose-dependent manner. TUNEL staining detects the apoptosis of primary neurons following treatment (a). Rh123 staining estimates the MMP of primary neurons after treatment (b). Scale bar: 20 μm. Data are mean ± SEM of four independent experiments for Kruskal–Wallis test, ##P < 0.01; for Bonferroni post hoc test (compare each group with control), ∗P < 0.05, ∗∗P < 0.01. [file Image_1.tif]

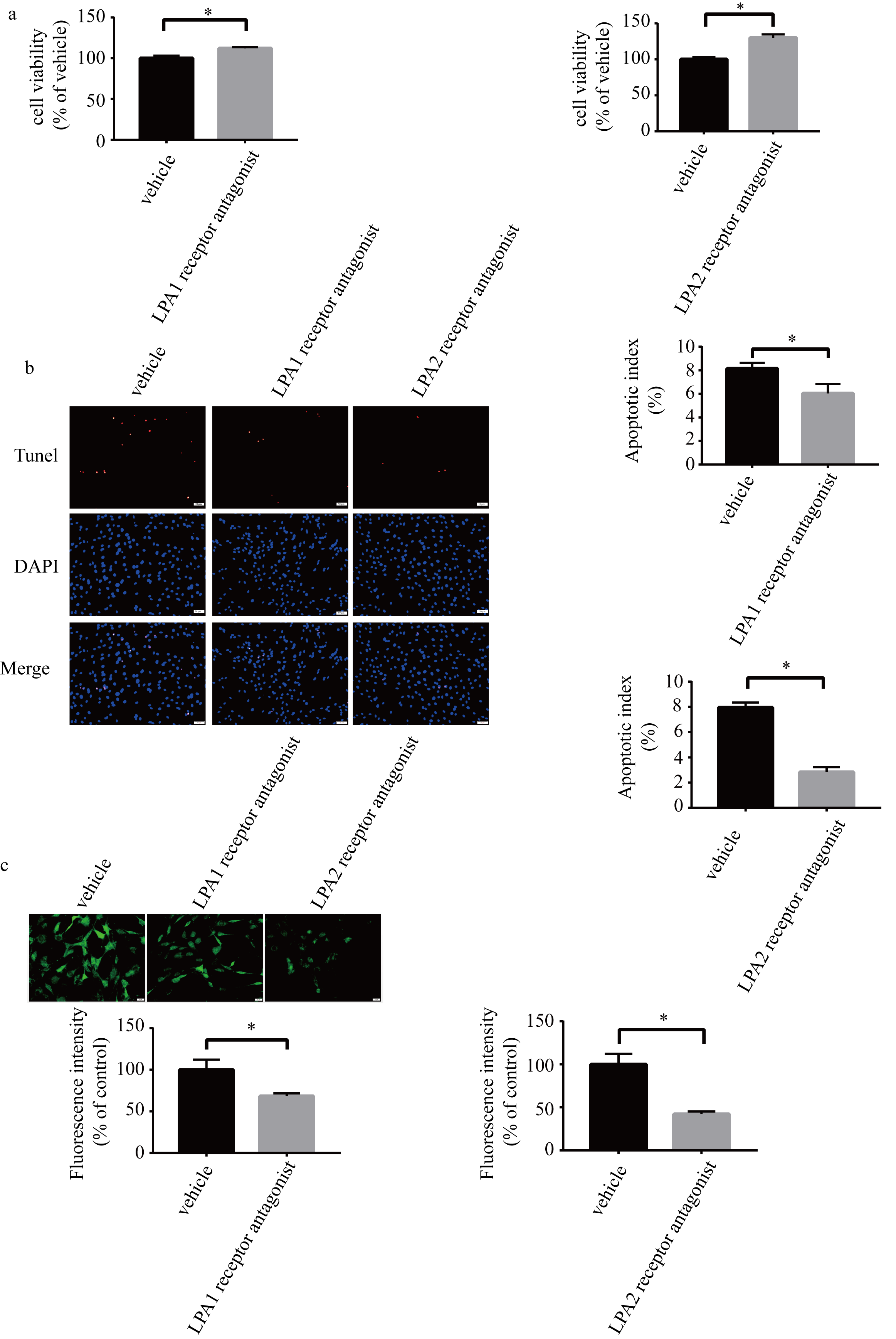

Supplement: FIGURE S2 — Blockade of LPA1 receptor and LPA2 receptor prevent LPA-induced neuronal damage as well as alleviating mitochondrial dysfunction. The viability of neuronal PC12 cells was measured with CCK-8 following treatment (a). The apoptosis of neuronal PC12 cells was detected by TUNEL staining after treatment. Scale bar: 50 μm. (b) The MMP of neuronal PC12 cells was estimated by Rh123 staining following treatment. Scale bar: 20 μm. (c) Data are mean ± SEM of four independent experiments. ∗P < 0.05, ∗∗P < 0.01. [file Image_2.tif]

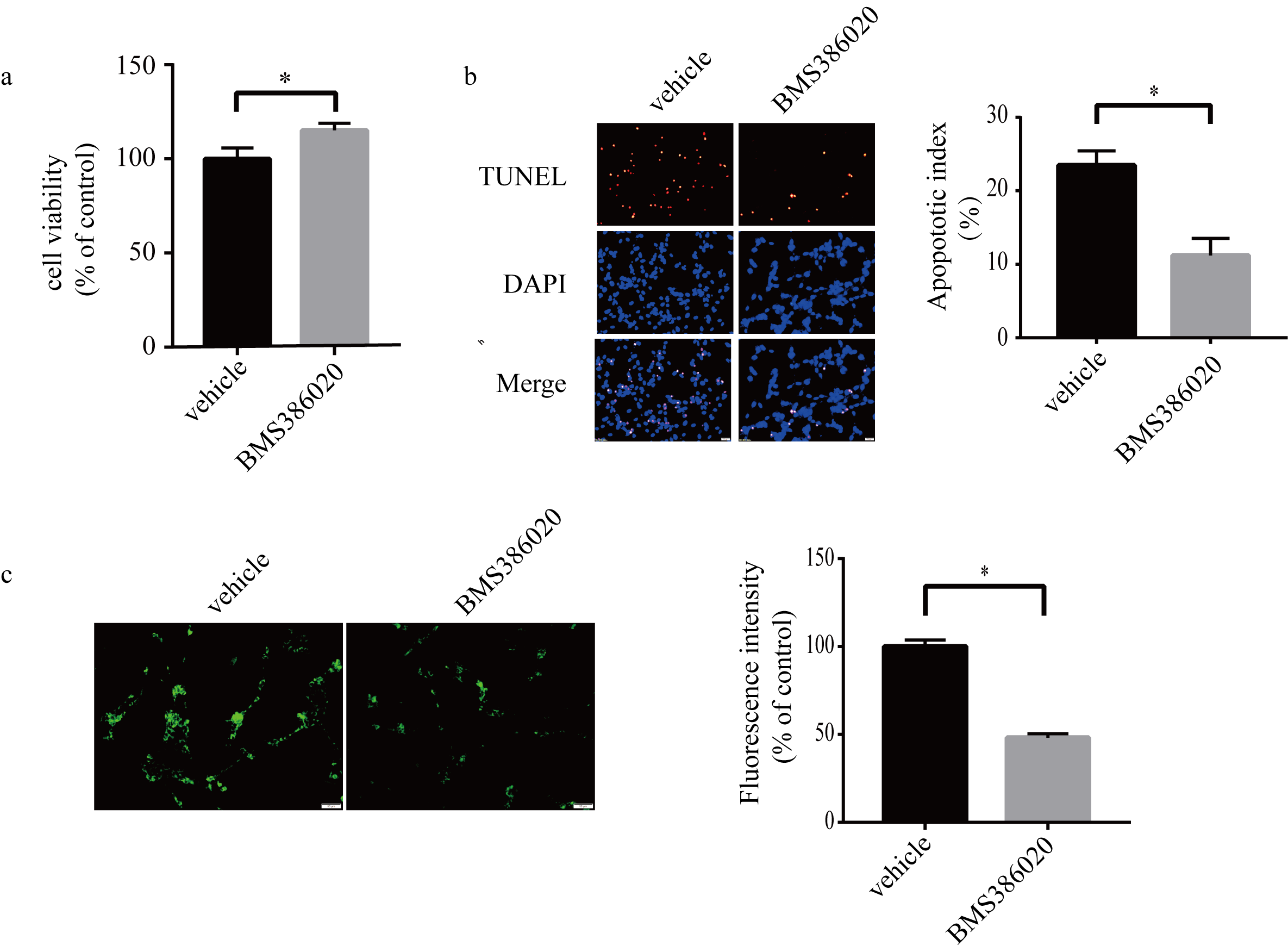

Supplement: FIGURE S3 — Blockade of LPA1 receptor using BMS986020 prevents LPA-induced neuronal damage and alleviates mitochondrial dysfunction. The viability of neuronal PC12 cells was measured using CCK-8 kit (a). The apoptosis of neuronal PC12 cells was detected by TUNEL staining. Scale bar: 20 μm. (b) The MMP of neuronal PC12 cells was estimated by Rh123 staining. Scale bar: 50 μm. (c) Data are mean ± SEM of four independent experiments. ∗P < 0.05, ∗∗P < 0.01. [file Image_3.tif]

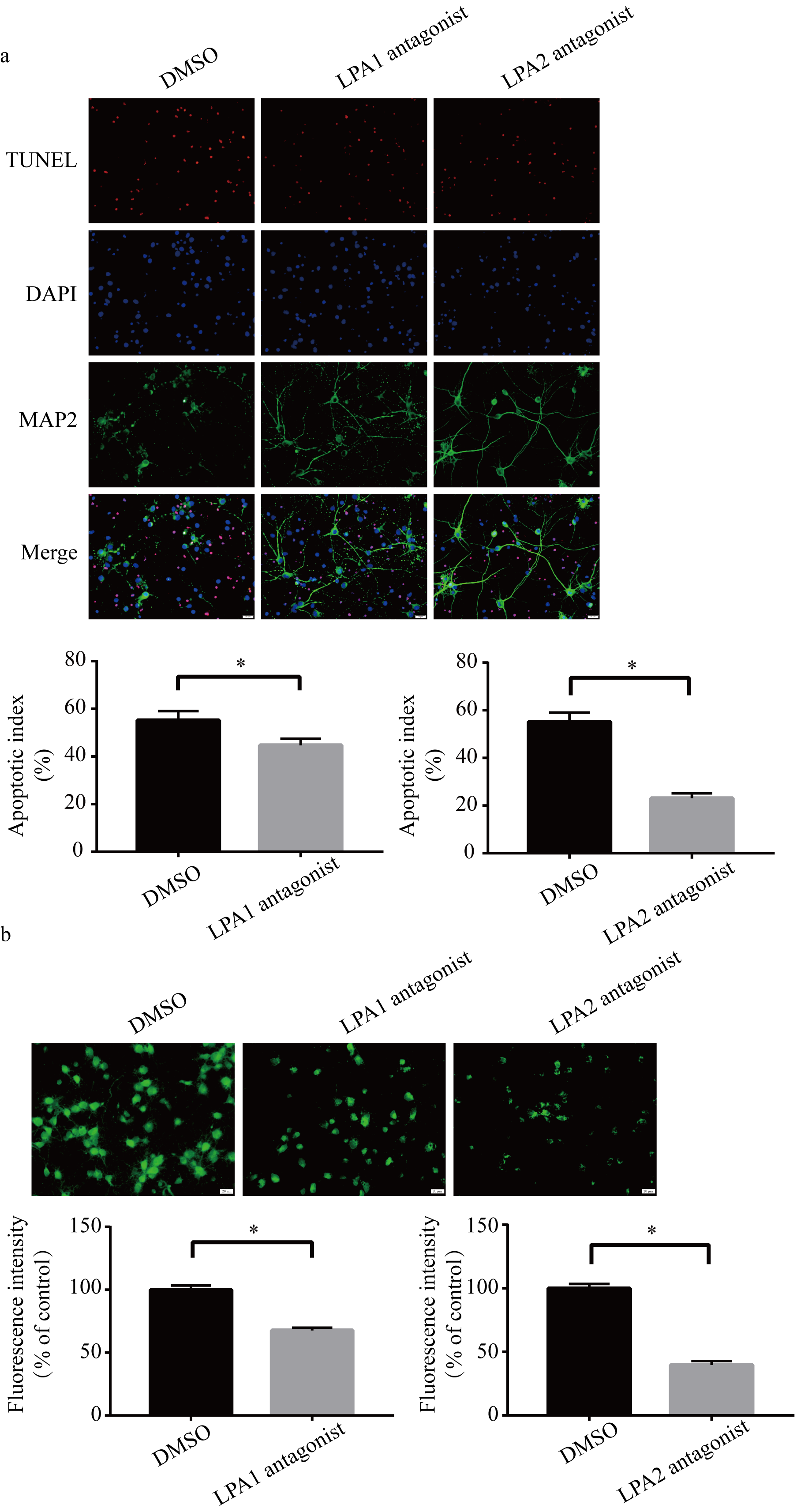

Supplement: FIGURE S4 — Blockade of LPA1 receptor and LPA2 receptor protects against LPA-induced neuronal damage, and alleviates mitochondrial dysfunction. The apoptosis of primary neurons was detected by TUNEL staining (a). The MMP of neuronal primary neurons was estimated by Rh123 staining (b). Scale bar: 20 μm. Data are mean ± SEM of four independent experiments. ∗P < 0.05, ∗∗P < 0.01. [file Image_4.tif]

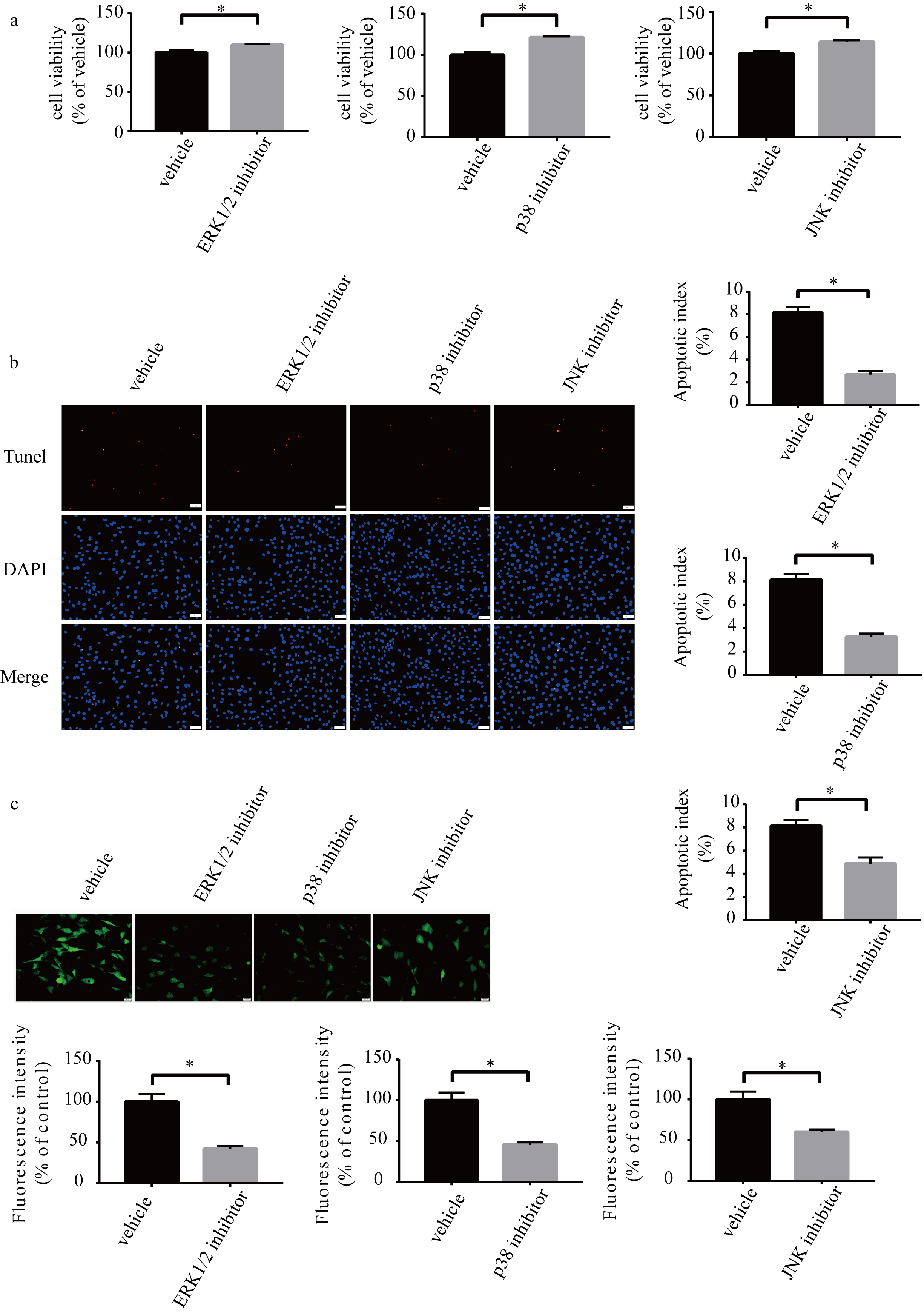

Supplement: FIGURE S5 — Blockade of MAPK pathway prevent LPA-induced neuronal damage as well as alleviating mitochondrial dysfunction. The viability of neuronal PC12 cells was measured with CCK-8 following treatment (a). The apoptosis of neuronal PC12 cells was detected by TUNEL staining after treatment. Scale bar: 50 μm (b). The MMP of neuronal PC12 cells was estimated by Rh123 staining following treatment. Scale bar: 20 μm. (c) Data are mean ± SEM of four independent experiments. ∗P < 0.05, ∗∗P < 0.01. [file Image_5.tif]

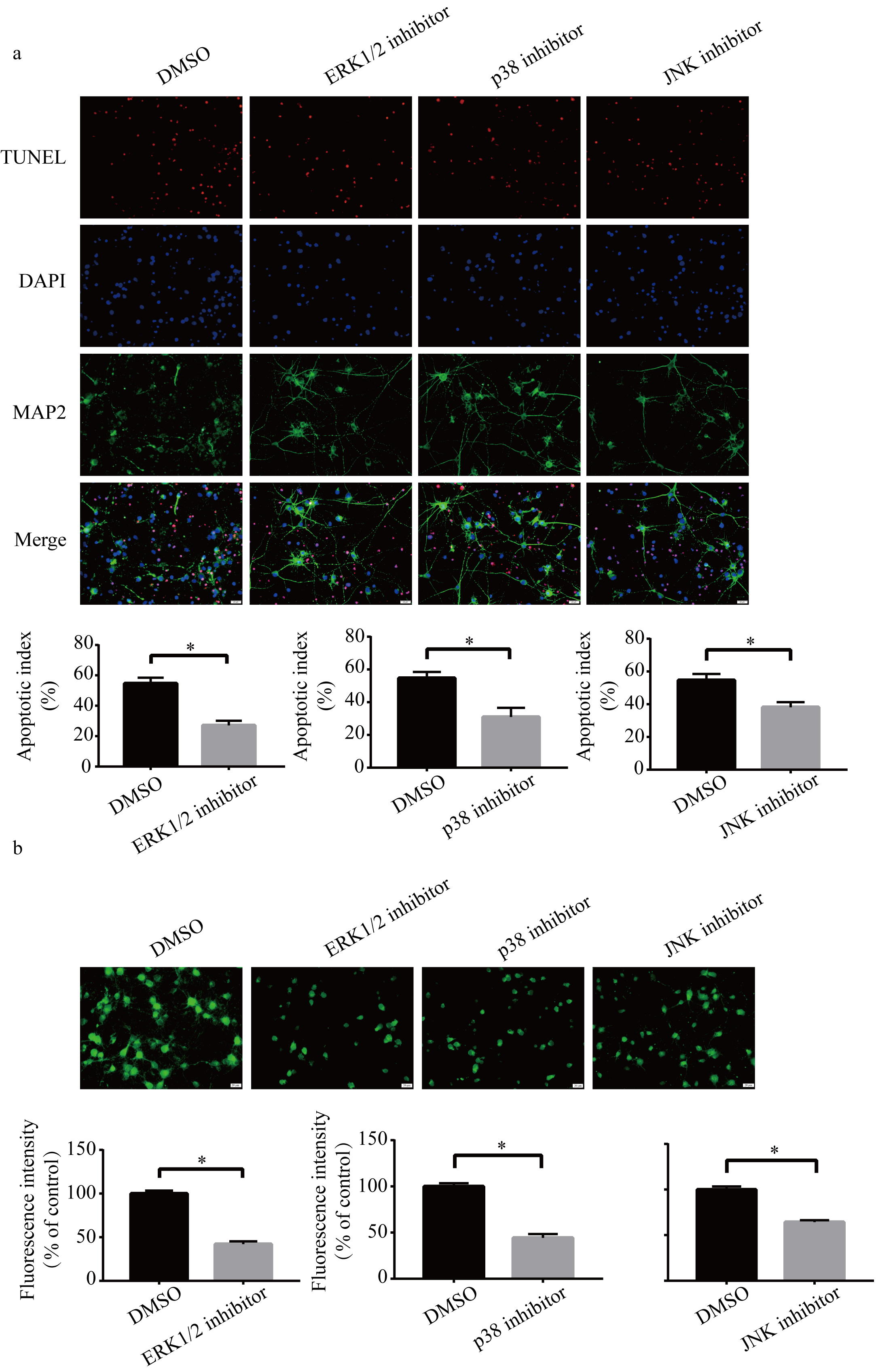

Supplement: FIGURE S6 — Blockade of MAPK pathway protects against LPA-induced neuronal damage, and alleviates mitochondrial dysfunction. The apoptosis of primary neurons was detected by TUNEL staining (A). The MMP of neuronal primary neurons was estimated by Rh123 staining (B). Scale bar: 20 μm. Data are mean ± SEM of four independent experiments. ∗P < 0.05, ∗∗P < 0.01. [file Image_6.tif]
